# Antimicrobial stewardship program prompts increased and earlier infectious diseases consultation

**DOI:** 10.1186/2047-2994-3-12

**Published:** 2014-04-17

**Authors:** Haley J Morrill, Melissa M Gaitanis, Kerry L LaPlante

**Affiliations:** 1 Providence Veterans Affairs Medical Center, Infectious Diseases Research Program, 830 Chalkstone Avenue, Providence, RI 02908, USA; 2 Department of Pharmacy Practice, College of Pharmacy, University of Rhode Island, 7 Greenhouse Road, Kingston, RI 02881, USA; 3 Division of Infectious Diseases, Warren Alpert Medical School of Brown University, 222 Richmond Street, Providence, RI 02903, USA

**Keywords:** Antimicrobial stewardship, Infectious diseases consult, Intervention

## Abstract

A recent analysis demonstrated that infectious diseases (ID) specialty intervention was associated with decreased mortality and hospital readmission. These benefits were greatest if involvement occurred within two days of hospital admission. Antimicrobial stewardship programs should augment the services of an ID specialist team and promote formal consultation. Implementation of an antimicrobial stewardship program at the Providence Veterans Affairs Medical Center was associated with an increased number of consults (increase of 72.2%) and decreased time to consult (3.5 days sooner), which might also dramatically improve patient outcomes, including mortality and readmission rates.

## Letters to the Editor

Dear Editor- We applaud Schmitt et al. [1] on their recent analysis which investigates the impact of infectious diseases (ID) consultation on patient outcomes using a national Medicare claims database. They found that intervention from ID specialists was associated with a 13% lower risk of 30-day mortality and 4% lower risk for readmission compared to no ID intervention. This strong association between ID involvement and improved patient outcomes highlights the importance of not only formal ID intervention but also coordinated antimicrobial stewardship efforts that augment the services of an ID consult team.

The benefits of antimicrobial stewardship in improving patient outcomes, decreasing costs, and limiting the emergence of resistance are widely recognized [2]. However, some institutions may be hesitant to implement antimicrobial stewardship programs (ASPs) due to a potential loss of ID consultations [3,4]. Nearly 50% percent of ID physicians believe that participation in ASPs leads to a decrease in requests for consultation [5]. ASPs should not interfere with the services of formal ID consult teams; rather ASPs should supplement the consult service and potentially generate ID consults requests [6].

At the Providence Veterans Affairs Medical Center, a small teaching institution licensed for 118 beds, we found that ID consults increased post-implementation of a formal ASP. Our ASP team provides prospective audit and feedback on all inpatient antimicrobial use (intravenous and oral) daily (Monday-Friday). In a retrospective analysis, we compared outcomes, including ID consults, between a pre-stewardship period (PSP; September 1, 2011 - August 31, 2012) and a stewardship period (SP; September 1, 2012 - August 31, 2013). The Wilcoxon Signed Rank test was used to compare mean monthly ID consults per 1000 patient days between the PSP and SP. We observed a significant 72.2% increase in ID consults per 1000 patient days from the PSP to the SP (p < 0.01).

Schmitt et al. found the benefits of a ID consult were greatest if the specialty service was consulted within 2 days of admission [1]. Early consultation was associated with significantly lower 30-day mortality and 30-day readmission, shorter length of stay in the hospital and intensive care unit, and decreased costs. Our daily prospective audit and feedback approach enables our ASP team members to efficiently identify complex patients with serious infections, who may benefit from formal ID assistance, early on in their treatment course. Post-implementation of our ASP, the mean time to ID consult from admission was 3.5 days sooner (time to consult; PSP - 7.4 ± 12.8 days versus SP - 3.8 ± 5.3 days).

We feel that antimicrobial stewardship programs have a significant potential to involve ID specialists in the care of patients with increasing numbers and sooner during hospitalization. Overall, our ASP increased the number of consults (increase of 72.2%) and decreased the time to consult (3.5 days sooner) within the first year of implementation, which when evaluated with the findings of Schmitt et al. could dramatically improve patient outcomes, including mortality and readmission rates.

## Abbreviations

ID: Infectious diseases; ASPs: Antimicrobial stewardship programs; PSP: Pre-stewardship period; SP: Stewardship period.

## Competing interests

The views expressed are those of the authors and do not necessarily reflect the position or policy of the United States Department of Veterans Affairs. This work was unfunded. Haley J. Morrill and Melissa M. Gaitanis have no conflicts to disclose. Kerry L. LaPlante has received research funding or acted as an advisor, speaker, or consultant for Astellas, Cubist, Forest, and Pfizer Inc.

## Authors’ contributions

HJM and KLL acquired and analyzed the data. HJM drafted the manuscript, while critical revision was provided by MMG and KLL. All authors read and approved the final version of the manuscript.
